# Rhizosphere microbiome dynamics and plant adaptation to abiotic stress in major oilseed crops: a review

**DOI:** 10.3389/fpls.2026.1832403

**Published:** 2026-05-25

**Authors:** Ainash Daurova, Dias Daurov, Zagipa Sapakhova, Rakhim Kanat, Zhanar Abilda, Maxat Toishimanov, Iskander Isgandarov, Almas Mukhametov, Dmitriy Volkov, Malika Shamekova, Kabyl Zhambakin

**Affiliations:** 1 Laboratory of Breeding and Biotechnology, Institute of Plant Biology and Biotechnology, Almaty, Kazakhstan; 2 Faculty of Agrobiology, Kazakh National Agrarian Research University, Almaty, Kazakhstan; 3 Tanir Research Laboratory LLP, Almaty, Kazakhstan

**Keywords:** abiotic stress, metagenomic, microbiome engineering, oilseed crops, omics technology, PGPR, rhizosphere microbiome, syncom

## Abstract

Abiotic stresses, such as drought, salinity, extreme temperatures, nutrient deficiencies, and heavy metal contamination, severely limit oilseed crop productivity under accelerating climate change. This review synthesizes recent advances in understanding the critical role of soil and plant-associated microbiomes in conferring stress tolerance to major oilseed species, including rapeseed (*Brassica napus*), sunflower (*Helianthus annuus*), soybean (*Glycine max*), and sesame (*Sesamum indicum*). Beneficial microorganisms, particularly plant growth-promoting rhizobacteria (PGPR), arbuscular mycorrhizal fungi (AMF), and endophytes, enhance plant tolerance through an integrated network of biochemical, physiological, and molecular mechanisms. Biochemically, they modulate phytohormone levels (e.g., IAA and ABA), produce osmoprotectants, and regulate antioxidant systems (e.g., SOD, CAT, POD) to mitigate oxidative damage. Physiologically, these processes contribute to improved root architecture, water-use efficiency, nutrient acquisition, and ion homeostasis under stress conditions. At the molecular level, microorganisms influence gene expression and signaling pathways associated with stress responses, including activation of stress-responsive genes and metabolic adjustments. These interconnected mechanisms collectively strengthen plant resilience by coordinating metabolic regulation, cellular protection, and adaptive responses within the plant-microbiome system. Agroecological practices (soil type, crop rotation, tillage, fertilization) strongly shape microbial community assembly and functional potential, while multi-omics approaches (metagenomics, metatranscriptomics, metabolomics) reveal stress-driven restructuring and adaptive metabolic shifts in the rhizosphere. Emerging tools such as synthetic microbial consortia (SynComs) and targeted microbiome engineering offer promising, sustainable alternatives to conventional breeding and chemical interventions, enhancing soil health, nutrient cycling, and agroecosystem resilience with reduced environmental footprint. This review presents a comprehensive synthesis with a specific focus on oilseed crops, integrating current knowledge on microbiome dynamics under multiple abiotic stress conditions—an area that remains comparatively underrepresented in the literature. It examines key microbial groups driving adaptation, evaluates omics-based insights into plant-microbiome interactions, identifies critical research gaps, and outlines future directions for microbial inoculants and climate-resilient oilseed production systems.

## 1 Introduction

Modern agriculture is undergoing significant changes driven by climate change and increasing human impact on ecosystems. Global warming is increasing the frequency and severity of extreme weather events, including droughts, floods, temperature fluctuations, and soil salinization, all of which pose major threats to crop productivity and long-term sustainability. Abiotic stresses are among the most significant constraints on global agricultural productivity and can lead to substantial yield losses, in some cases exceeding 50% under severe environmental conditions, with heat, salinity, heavy metals, and water deficits being primary contributors (Salam et al., 2023). Even a modest 1 °C rise in temperature can reduce yields of major crops by 3–7%, further aggravating global food security concerns (Zhao et al., 2017). With the global population projected to surpass 10 billion by mid-century, enhancing crop productivity has become imperative (Moore et al., 2022). Yet dependence on synthetic fertilizers raises serious environmental and economic issues.

These stresses impair not only plant physiological processes, such as photosynthesis, water relations, and nutrient uptake, but also alter soil physicochemical properties, including pH, moisture content, salinity levels, and mineral availability, which in turn directly compromise crop growth and yield (Zhang et al., 2020). Soil salinity, for example, induces osmotic stress, disrupts ion homeostasis, and inhibits root development, while heavy metal accumulation exerts toxic effects that interfere with essential biochemical pathways (Sridhar et al., 2025a). Oilseed crops, including rapeseed (*Brassica napus*), soybean (*Glycine max*), sunflower (*Helianthus annuus*), and sesame (*Sesamum indicum*), are particularly susceptible, with yields potentially declining by up to 70% under elevated rhizosphere concentrations of sodium and chloride (Joshi et al., 2021).

Conventional approaches to mitigating abiotic stress in oilseed crops rely heavily on classical breeding, genetic engineering, and chemical soil amendments (Daurova et al., 2020b). However, breeding for drought and salinity tolerance in species such as *B. napus* and *H. annuus* is constrained by the polygenic nature of stress-related traits and limited genetic variation, resulting in prolonged selection cycles. Genetic engineering, while promising, faces significant regulatory, biosafety, and public acceptance challenges in many regions (Afzal et al., 2023). Chemical amendments, costs andypsum for sodic soils or chelators for heavy metal immobilization, often require repeated applications, increase production costs, and may contribute to secondary soil degradation and microbial imbalance (Afzal et al., 2023).

In contrast, microbiome-based strategies offer a complementary and potentially more sustainable approach by leveraging naturally occurring plant-microbe interactions. Unlike conventional methods that primarily target plant traits or soil chemistry, microbiome-centered approaches enhance stress tolerance through dynamic, multi-functional mechanisms, including improved nutrient acquisition, phytohormone regulation, and stress-responsive metabolic adjustments. These strategies can be more adaptable to environmental variability and may contribute to long-term soil health and agroecosystem resilience, thereby addressing some of the limitations associated with traditional approaches.

Considering these limitations, increasing attention has turned to biological strategies that exploit the soil microbiome to bolster plant stress tolerance. Contemporary research underscores the critical role of soil- and plant-associated microbiomes in mitigating abiotic stress impacts. Microorganisms in the rhizosphere, phyllosphere, and endosphere establish complex mutualistic interactions with plants, improving nutrient acquisition, synthesizing phytohormones, detoxifying harmful compounds, and conferring protection against pathogens (Wu and Bose, 2024; Verma et al., 2021).

These interactions are mediated through specific molecular and biochemical mechanisms, including root exudate-driven recruitment of beneficial microbes and bidirectional signaling between plants and microorganisms. Root-secreted compounds such as sugars, amino acids, and secondary metabolites selectively shape microbial community composition, while associated microbes, in turn, modulate plant signaling pathways through phytohormone crosstalk (e.g., auxin-ABA-ethylene interactions) and activation of stress-responsive gene networks. In addition, microbial traits such as ACC deaminase activity, siderophore production, and exopolysaccharide synthesis contribute to stress mitigation by regulating ethylene levels, improving nutrient availability, and enhancing rhizosphere stability under adverse conditions.

Diverse bacterial genera, including *Azotobacter*, *Azospirillum*, *Rhizobium*, *Bacillus*, and *Pseudomonas*, enhance crop resilience to drought, salinity, and other stressors (Dhungana et al., 2023; Hartman et al., 2023).

Phytomicrobiome studies are especially relevant for oilseed crops, which occupy extensive arable land and are vital for food security and economic stability in numerous countries (Khan et al., 2024). Plants assemble diverse microbial communities from soil, seeds, parental tissues, and the environment; these communities, transmitted vertically and horizontally, support early development and modulate stress responses. In fluctuating environments, they actively facilitate adaptation by regulating gene expression and metabolic pathways, thereby strengthening stress resistance (Sagar et al., 2021).

In key oilseed species such as rapeseed (*B. napus*) and sunflower (*H. annuus*), plant growth-promoting rhizobacteria (PGPR) belonging to genera including *Alcaligenes*, *Acinetobacter*, *Pseudomonas*, *Azospirillum*, and *Bacillus* alleviate salinity- and drought-induced stress by restricting sodium uptake, improving potassium retention, promoting root architecture, and activating antioxidant enzyme systems (Guarino et al., 2020; Zaib et al., 2023). These PGPR further mitigate toxic ion accumulation, restore nutrient homeostasis, and reinforce plant defense mechanisms (Li et al., 2023a).

Consequently, microbiome-centric approaches are gaining prominence for reducing chemical inputs while improving resilience to extreme conditions (Lal et al., 2021; O’Callaghan et al., 2022). Recent evidence demonstrates that microbial bioinoculants and biostimulants effectively shield plants from abiotic stresses (Ali et al., 2024; Sridhar et al., 2025b), while synthetic microbial communities (SynComs) enable targeted engineering of microbiota to optimize nutrient uptake and adaptation to salinity and drought (Schmitz et al., 2022; Muhammad et al., 2025; Buqori et al., 2025). Microbial technologies thus emerge as a cornerstone of sustainable agriculture, supporting higher yields with reduced environmental footprint.

The objective of this review is to provide an integrative synthesis of current knowledge on the structure and function of microbiomes associated with oilseed crops under abiotic stress conditions. We adopt a conceptual framework in which the plant-associated microbiome is viewed as a dynamic and adaptive system that mediates plant responses to environmental stress through interconnected biochemical, physiological, and molecular processes.

Within this framework, the review examines how major abiotic stressors including drought, salinity, temperature extremes, nutrient deficiencies, and heavy metals reshape microbial community composition and function, and how these changes influence plant stress tolerance. Emphasis is placed on the roles of rhizosphere, endophytic, and phyllosphere microbiomes, as well as on insights derived from modern omics approaches. The review also highlights key knowledge gaps and outlines future directions for the development of microbiome-based strategies for sustainable and climate-resilient oilseed production.

Despite the growing body of literature on plant-microbiome interactions, evidence specifically derived from oilseed cropping systems remains limited. Consequently, many conclusions presented in this review are extrapolated from studies conducted in model or non-oilseed plant systems, highlighting the need for cautious interpretation and further crop-specific investigation.

## 2 Literature search and study selection strategy

To ensure a comprehensive, objective, and up-to-date synthesis of the available scientific evidence, a structured literature search strategy was conducted using major international databases, including Web of Science, Scopus, and PubMed. The search covered the period from 2000 to 2026, with particular emphasis on studies published within the last 10–16 years that reflect recent advances in plant-microbiome interactions under abiotic stress conditions.

Relevant studies were identified using carefully designed combinations of English-language keywords and phrases, including *“rhizosphere microbiome”, “oilseed crops”, “abiotic stress”, “drought”, “salinity”, “plant growth-promoting rhizobacteria (PGPR)”, “arbuscular mycorrhizal fungi (AMF)”, “microbiome engineering”, “synthetic microbiome”, “omics technologies”, “metagenomics”, “metatranscriptomics”*, and *“plant–microbe interactions”*. Search queries were constructed using Boolean operators (AND, OR, NOT) to maximize both precision and coverage.

At the initial stage, titles and abstracts of all retrieved publications were screened for relevance. Potentially relevant articles were subsequently subjected to full-text evaluation. Additional sources were identified through manual screening of reference lists in key review articles, highly cited publications, and relevant monographs (snowballing approach). This strategy enabled the inclusion of important studies that may not have been captured through automated database searches alone.

Inclusion and exclusion criteria. Only peer-reviewed research articles and review papers published in English were considered. Studies included if they addressed microbial community structure, functional mechanisms, and the role of microbiota in the adaptation of oilseed crops (or closely related plant systems) to abiotic stress factors. Particular attention was given to studies providing clear methodological descriptions, employing modern molecular and omics approaches, and demonstrating practical or ecological relevance.

Non-peer-reviewed sources (e.g., preprints and conference abstracts without subsequent publication), studies with insufficient methodological detail, and articles focusing exclusively on model plant systems without relevance to oilseed crops were excluded. Additionally, studies not addressing plant-microbiome interactions under stress conditions, as well as duplicate or outdated publications with limited scientific value, were not considered.

**Figure 1:**
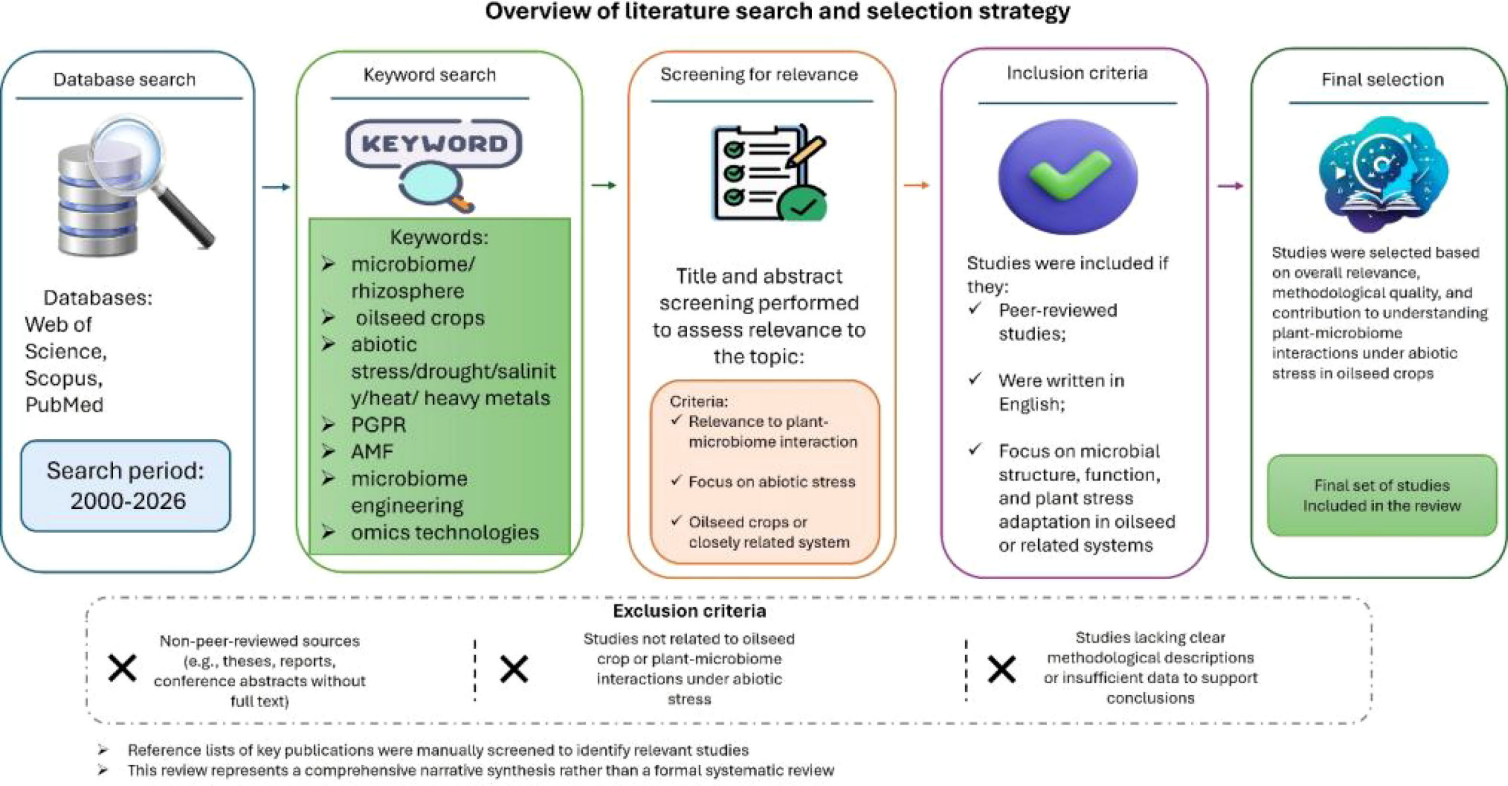
Conceptual workflow of the literature search and study selection process, including database search, keyword strategy, screening procedures, and inclusion/exclusion criteria used in this narrative review.

This work represents a comprehensive narrative review rather than a formal systematic review or meta-analysis. Nevertheless, efforts were made to ensure transparency, reproducibility, and balanced coverage of the literature. The literature search strategy and study selection process are summarized in **Figure 1**.

This approach enabled the integration of both fundamental mechanisms of plant-microbiome interactions and applied aspects of microbiome-based strategies, providing a comprehensive overview of the current state of research in this field.

## 3 Soil microorganisms and associations with oilseed crops

The soil microbial community is a fundamental component of agroecosystems, particularly under conditions of abiotic stress such as drought, salinity, and temperature fluctuations, which are common in regions with sharply continental climates. A comprehensive understanding of the composition, functional roles, and plant interaction mechanisms of the soil microbiome, especially in strategically important oilseed crops such as rapeseed, sunflower, and soybean is essential for developing resilient and sustainable agricultural systems. **Table 1** summarizes the dominant microbial groups associated with these major oilseed crops and highlights their key functional roles in nutrient cycling, stress tolerance, and plant growth promotion, clearly illustrating the crop-specific nature of microbiome assembly. The soil microbiome forms a highly dynamic and functionally intricate system that profoundly influences oilseed crop performance under abiotic stress conditions.

**Table 1:** Representative soil microorganisms, their ecological roles, and associations with oilseed crops.

Microorganism	Associated oilseed crops	Functional role in soil and plant interaction	References
*Bacillus* spp.*, Pseudomonas* spp.	*B. napus, H. annuus*, *G. max*	Organic matter decomposition; phosphate solubilization; phytohormone production; biocontrol	(Gouda et al., 2018; Egamberdieva et al., 2019)
*Azotobacter* spp.*, Azospirillum* spp.	*B. napus, Camelina sativa, H. annuus*	Nitrogen fixation; root growth promotion; improved nutrient uptake	(Rojas-Tapias et al., 2012)
*Rhizobium* spp.*, Bradyrhizobium* spp.	*G. max* (primary host); benefits *B. napus* in rotation	Symbiotic nitrogen fixation; improved soil N availability	(Mus et al., 2016)
*Glomus* spp.*, Rhizophagus irregularis*	*B. napus, H. annuus, C.sativa*	Phosphorus uptake; drought tolerance; root development	(Begum et al., 2019)
*Endophytic bacteria (Enterobacter* spp.*, Burkholderia* spp.*, Pantoea* spp.*)*	*G. max, B. napus*	Endophytic colonization; phytohormone production; stress tolerance; pathogen suppression	(Kandel et al., 2017; Santoyo et al., 2016)
*Actinomycetes (Streptomyces* spp.*)*	*H. annuus, B. napus*	Lignocellulose degradation; antibiotic production; soil organic matter formation	(Bhatti et al., 2017)
Halotolerant/drought-tolerant rhizobacteria (*Halomonas* spp., *Paenibacillus* spp., *Bacillus halotolerans*)	*C. sativa, B. napus, H. annuus*	Osmotic regulation; antioxidant activation; salinity and drought tolerance	(Egamberdieva et al., 2019)

Recent microbiome research indicates that major oilseed crops shape distinct microbial communities within their rhizosphere and surrounding soil environment through the selective effects of root exudates and plant genotype (Yao et al., 2026; Santoyo et al., 2016; Lovecká et al., 2023). Comparative analyses of microbial assemblages associated with rapeseed (*B. napus*), sunflower (*H. annuus*), soybean (*Glycine max*), and sesame (*Sesamum indicum*) demonstrate that oilseed crops consistently recruit several dominant bacterial groups, including members of the genera *Pseudomonas, Bacillus, Enterobacter, Pantoea, and Sphingomonas*, as well as actinobacterial taxa such as *Curtobacterium*, *Microbacterium*, and *Streptomyces* (Santoyo et al., 2016; Kandel et al., 2017; Isgandarov et al., 2026). These microorganisms contribute to key ecological functions including nutrient mobilization, phytohormone production, suppression of soil-borne pathogens, and improved plant tolerance to environmental stressors (Backer et al., 2018; Gouda et al., 2018).

Importantly, the structure of microbial communities varies among oilseed crop species. Rapeseed rhizospheres are frequently enriched with *Pseudomonas* and *Bacillus* species associated with phosphorus solubilization and pathogen suppression (Gouda et al., 2018; Egamberdieva et al., 2019), whereas sunflower soils often harbor higher abundances of actinobacteria such as *Streptomyces*, which contribute to organic matter decomposition and antibiotic production (Bhatti et al., 2017; Newitt et al., 2019). In contrast, soybean-associated microbial communities are strongly influenced by symbiotic nitrogen-fixing bacteria including *Rhizobium* and *Bradyrhizobium*, reflecting the legume-specific capacity for biological nitrogen fixation (Mus et al., 2016). These crop-specific microbial assemblages highlight the importance of host-driven microbiome assembly and suggest that oilseed cropping systems may represent potential sources of beneficial microorganisms for microbiome-based agricultural strategies, although further crop-specific validation is required (Dwivedi et al., 2025).

### 3.1 Bacteria

Bacteria constitute the most abundant and diverse microbial group in soil, forming the cornerstone of its biogeochemical functionality, with populations reaching up to 10 billion cells per gram and encompassing thousands of species. They orchestrate critical cycles of nitrogen, phosphorus, and carbon, facilitate organic matter degradation, and provide protection against phytopathogens (Santoyo et al., 2016; Lovecká et al., 2023; Ma et al., 2016). Dominant phyla, including *Proteobacteria*, *Actinobacteria*, *Acidobacteria*, *Chloroflexi*, and *Nitrospirae*, predominate across varied soil environments (Batista et al., 2018).

In oilseed cropping systems, however, the functional roles of bacteria exhibit crop-specific patterns. For example, in *B. napus* (rapeseed), members of *Pseudomonas* and *Bacillus* are frequently associated with phosphorus solubilization, pathogen suppression, and enhanced tolerance to salinity and drought. In sunflower (*Helianthus annuus*), actinobacterial taxa such as *Streptomyces* contribute to organic matter turnover and production of antimicrobial compounds, while in soybean (*Glycine max*), symbiotic nitrogen-fixing bacteria such as *Rhizobium* and *Bradyrhizobium* play a central role in nitrogen acquisition and plant growth. These crop-specific associations highlight the variability of bacterial functions across oilseed systems and underscore the importance of host microbiome interactions in shaping stress adaptation.

Bacteria execute diverse functions essential for plant health. Nitrogen-fixing species such as *Rhizobium* and *Azotobacter* convert atmospheric N_2_ into forms accessible to plants, a process particularly vital for legumes in regions with sharply continental climates (Gage et al., 2018). Phosphate-mobilizing bacteria like *Pseudomonas* and *Bacillus* render insoluble phosphates bioavailable (Sharma et al., 2019). Plant growth-promoting rhizobacteria (PGPR) contribute to plant performance through multiple mechanisms, including phytohormone production (e.g., auxins), modulation of plant signaling pathways, and suppression of phytopathogens. These interactions can enhance plant growth and improve tolerance to abiotic stresses such as drought, although their effectiveness is context-dependent and varies across plant species and environmental conditions (Dwivedi et al., 2025; Backer et al., 2018). Field studies, for instance, demonstrate that bacterial inoculant concentration and soil fertility modulate endophytic colonization by *Bacillus subtilis*, influencing growth promotion in soybean and maize (Bueno et al., 2022).

Soil pH exerts a profound influence on bacterial community structure, *Acidobacteria* grow in acidic conditions (pH <6), while *Proteobacteria* favor neutral pH (Wang et al., 2022). Parameters such as humidity and organic matter content further modulate composition. Crop rotation and minimal tillage practices foster bacterial diversity, whereas intensive agriculture diminishes it (Wang et al., 2025a). In low-organic-matter soils under moderate drought, rhizobacterial inoculation of oilseeds has been reported to improve plant productivity by enhancing root architecture, nutrient acquisition, chlorophyll content, and antioxidant defense systems.

### 3.2 Fungi

Soil fungi rank as the second most prevalent microbial group, playing indispensable roles in organic matter decomposition and soil aggregate formation as saprotrophs, pathogens, or symbionts such as mycorrhizal fungi that facilitate nutrient acquisition (da Silva et al., 2024; Tedersoo et al., 2020). Principal phyla, *Ascomycota*, *Basidiomycota*, and *Zygomycota* exhibit adaptations to diverse soil conditions and functional versatility (Jiao and Lu, 2020). Saprotrophic fungi such as *Penicillium* and *Aspergillus* degrade recalcitrant compounds including lignin and cellulose, thereby contributing to carbon cycling and nutrient availability for plants (Mayer et al., 2021).

Mycorrhizal fungi, notably *Glomus* species, establish symbiotic associations with roots, enhancing water and phosphorus uptake, which is particularly important for oilseed crops in water-limited environments (Qi et al., 2022; Smith and Wan, 2019). These fungi also promote soil aggregation, improving water retention and mitigating erosion (Smith and Wan, 2019). Biocontrol agents such as *Trichoderma* antagonize pathogens like *Fusarium*, thereby reducing disease incidence in crops including sunflower (Luo et al., 2023).

However, the structure and functional roles of fungal communities are highly context-dependent and influenced by environmental conditions and crop systems. Factors such as soil moisture, pH, temperature, and agricultural practices strongly affect fungal diversity, colonization efficiency, and symbiotic performance. For instance, arid conditions often favor drought-tolerant taxa such as *Aspergillus* (Niaz et al., 2024), while crop rotation generally enhances fungal diversity compared to monocropping systems (Šeremešić et al., 2024).

Crop-specific responses further contribute to this variability. In sunflower, mycorrhizal associations are often linked to improved phosphorus uptake and stress tolerance, whereas in *Brassica* species, which are typically non-mycorrhizal or weakly mycorrhizal, fungal contributions may be more indirect and related to soil processes and pathogen suppression. Applications of mycorrhizal and *Trichoderma*-based preparations have been reported to improve plant performance and reduce reliance on chemical inputs, although their effectiveness varies depending on local edaphic conditions and management practices (Vincze et al., 2024). Metagenomic approaches further support the selection of stress-adapted fungal strains, particularly for use in arid and marginal environments (Tedersoo et al., 2020).

### 3.3 Archaea

Archaea comprise a numerically minor yet functionally critical component of soil microbiomes, particularly in nitrogen biogeochemical cycling. Ammonia-oxidizing archaea (AOA) within *Thaumarchaeota* dominate nitrification in low-nitrogen or stressed soils (Khan et al., 2025b; Miltner et al., 2020). In nitrogen-limited rapeseed systems, archaeal ammonium oxidation may prevail over bacterial pathways, directly influencing nitrate availability and crop productivity (Cheng et al., 2022). *Euryarchaeota* methanogens contribute to carbon cycling in anaerobic, waterlogged soils (Kercheva et al., 2025).

Archaeal community structure correlates strongly with soil physicochemical properties: *Thaumarchaeota* tend to dominate in acidic environments, whereas *Euryarchaeota* are more abundant in alkaline conditions (Labouyrie et al., 2023). Organic farming practices can increase archaeal abundance, while excessive nitrogen fertilization may suppress their activity (Miltner et al., 2020). Although adjustments in soil pH and nutrient management have the potential to influence archaeal-driven nitrification processes, the magnitude of these effects varies depending on environmental conditions and soil characteristics.

Despite advances in metagenomic approaches, archaeal ecology remains less understood than that of bacteria and fungi, which limits their current application in agriculture (Khan et al., 2025b; Kercheva et al., 2025). Future research should focus on elucidating the functional diversity and ecological roles of archaeal communities across different oilseed cropping systems, as well as their interactions with plant roots and other microbiome components. Greater integration of omics technologies may help identify key archaeal taxa and metabolic pathways relevant to nutrient cycling and stress adaptation. In the long term, this knowledge could support the development of microbiome-based strategies that incorporate archaeal functions to optimize nitrogen use efficiency, reduce fertilizer inputs, and enhance agroecosystem sustainability under abiotic stress conditions.

Although these microbial functions are well documented across a wide range of plant systems, their specific roles and interactions in oilseed crops are less comprehensively characterized. This gap underscores the importance of expanding oilseed-focused microbiome studies under diverse environmental conditions.

### 3.4 Viruses

Despite their vast abundance in soil, viruses remain one of the least studied components of the microbial community. Viruses, predominantly bacteriophages infecting bacteria and archaea, occur in extremely high numbers in soil and influence microbial dynamics through cell lysis and horizontal gene transfer (Smith and Wan, 2019). These processes contribute to nutrient recycling (e.g., carbon and nitrogen release) and can shape microbial adaptation to environmental stress via the dissemination of functional genes. Viral abundance generally increases in organic-rich soils with high microbial density (Cheng et al., 2022), whereas intensive tillage practices may reduce viral diversity, while organic management systems tend to promote it.

In agricultural contexts, beneficial soil microorganisms, including actinomycetes, have been explored as biocontrol agents and plant growth promoters, contributing to improved plant productivity and soil health (AbdElgawad et al., 2020). However, their practical application remains limited by several factors, including host specificity, environmental instability, and challenges related to formulation and field-level persistence. In addition, the complexity of soil ecosystems and interactions with native microbiota complicate the predictability of phage-based interventions.

Current research is therefore focused on improving our understanding of soil viral ecology, host–phage interactions, and the role of viruses in shaping microbiome structure and function. Advances in metagenomics and viromics are beginning to reveal viral diversity and functional potential, yet significant knowledge gaps remain. Addressing these limitations will be essential for developing reliable and scalable virus-based strategies for sustainable crop protection under abiotic stress conditions.

### 3.5 Actinomycetes

Actinomycetes represent a unique bacterial group with fungus-like traits, forming mycelia that decompose complex organics like chitin, cellulose, and lignin. They can form mycelium, which plays an important role in the decomposition of complex organic compounds such as chitin, cellulose, and lignin (Bhatti et al., 2017; AbdElgawad et al., 2020). Members of the genus *Streptomyces* constitute a significant proportion of soil bacteria, reaching up to 10% (Zappelini et al., 2018). These microorganisms secrete enzymes (e.g., cellulases and chitinases) that accelerate the decomposition of plant residues and improve soil structure. They also synthesize antibiotics such as streptomycin and tetracycline, which inhibit pathogen growth (Silva et al., 2022).

In intensive farming environments, where agroecosystems are at risk from phytopathogens, actinomycetes can serve as an effective biological control agent. For example, *Streptomyces* griseus exhibits activity against phytopathogenic fungi such as *Fusarium*, resulting in a 20-25% reduction in the incidence of sunflower and rapeseed diseases (Newitt et al., 2019). The composition of actinomycete microbial communities depends on soil conditions; they prefer soils rich in organic matter and thrive under crop rotation (Zhao et al., 2025a).

The development of actinomycete-based biofungicides and biostimulants is a promising area. Seed treatment with *Streptomyces* can increase oilseed yields by 10-15% (Vincze et al., 2024). For successful application in arid regions where oilseed crops are grown, drought-resistant actinomycete strains must be selected (Kovaleski et al., 2025). Metagenomic and biotechnological approaches offer new opportunities for the use of these microorganisms in sustainable agriculture (AbdElgawad et al., 2020; Grover et al., 2016; Isgandarov et al., 2026).

The soil microbiome, encompassing bacteria, fungi, archaea, viruses, and actinomycetes, integrally sustains soil fertility, plant nutrition, pathogen defense, and growth regulation under abiotic stress. Leveraging this knowledge fosters innovative, climate-resilient strategies for oilseed production in continental agroecosystems.

## 4 Abiotic stress and soil microbial communities’ structure of oilseeds

Abiotic stresses, including drought, salinity, thermal fluctuations, and heavy metal pollution, significantly affect the structure, biodiversity, and functional activity of soil microbial communities. These impacts alter the physical and chemical properties of the soil, as well as the availability of water and nutrients, thereby influencing bacterial and fungal populations, particularly in the plant rhizosphere (Ebrahimi-Zarandi et al., 2023).

Abiotic stress factors, including drought, salinity, temperature extremes, and heavy metal contamination, reshaping soil physicochemical properties and drive shifts in microbial community composition and functional potential in the rhizosphere (Ebrahimi-Zarandi et al., 2023). These changes often result in the enrichment of stress-tolerant taxa, such as *Actinobacteria*, *Firmicutes*, and other halotolerant or metal-resistant microorganisms, capable of sustaining key ecological functions under adverse conditions.

**Figure 2:**
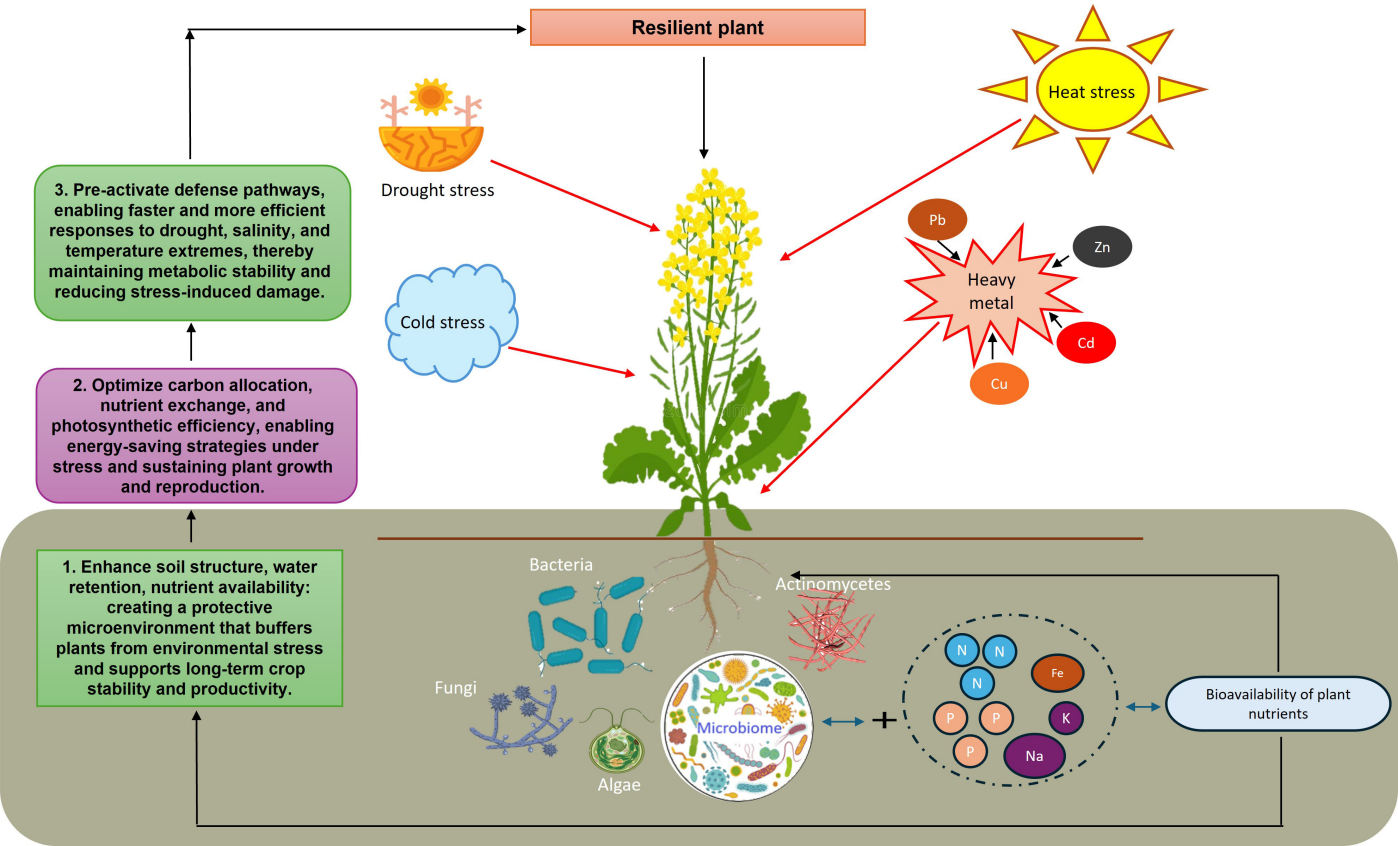
Conceptual model illustrating abiotic stress - driven restructuring of the oilseed rhizosphere microbiome and its role in plant stress tolerance.

The following sections focus on how these stress-induced shifts in microbial communities influence the structure, diversity, and functional dynamics of oilseed-associated microbiomes. Collectively, these microbiome-mediated processes improve nutrient bioavailability, stabilize plant metabolism, and contribute to enhanced stress tolerance in oilseed cropping systems (**Figure 2**). **Figure 2** illustrates the key microbiome-mediated mechanisms involved in plant adaptation to abiotic stress, integrating biochemical, physiological, and molecular pathways discussed in this section.

### 4.1 Drought

Drought represents one of the most severe abiotic constraints affecting oilseed production worldwide (Ebrahimi-Zarandi et al., 2023; Sharma et al., 2025; Gupta et al., 2020). Beyond its direct impact on plant physiology, manifested through reduced cell turgor, impaired photosynthesis, inhibited cell division, and decreased nutrient uptake (Naylor et al., 2017; Vurukonda et al., 2016; Zboralski and Filion, 2023), water deficit substantially alters soil physicochemical properties, including moisture content, oxygen diffusion, nutrient mobility, and osmotic potential. These changes directly reshape rhizosphere microbial community structure and functional activity.

Several studies in *B. napus* rhizospheres demonstrate that drought reduces total microbial biomass and alters taxonomic composition (Xu et al., 2018; Xie et al., 2021). Typically, the relative abundance of *Proteobacteria* and *Bacteroidetes* declines, whereas drought-tolerant taxa such as *Actinobacteria* and *Firmicutes* become enriched. These groups possess adaptive traits including spore formation, thick cell walls, osmoprotectant synthesis, and efficient resource utilization under low-moisture conditions.

However, findings across studies are not entirely consistent. While some reports indicate a pronounced enrichment of *Actinobacteria* under drought conditions, others show more moderate shifts or context-dependent responses, particularly in soils with higher organic matter or irrigation history. Such variability reflects differences in soil texture, nutrient availability, climatic conditions, crop genotype, and stress intensity or duration. In addition, plant-mediated effects, including changes in root exudation patterns under drought, can further influence microbial recruitment and community restructuring.

These contrasting observations suggest that drought-induced microbiome responses are highly context-specific and cannot be generalized across all oilseed systems, emphasizing the need for site-specific and system-level interpretations (Lavallee et al., 2024). The response of microbial communities to drought is further modulated by soil type, plant genotype, and stress duration, leading to substantial variability across studies. Soil texture and structure play a critical role: sandy soils, characterized by low water-holding capacity, often exhibit more pronounced reductions in microbial biomass and stronger selection for drought-tolerant taxa, whereas clay-rich soils can buffer moisture fluctuations and support more stable microbial communities. Soil organic matter content also influences microbial resilience by providing substrates that sustain microbial activity under water-limited conditions.

Plant genotype represents another key determinant of microbiome response. Different oilseed cultivars vary in root architecture and exudation profiles, which influence microbial recruitment and community restructuring under drought. Genotypes with deeper or more extensive root systems may maintain more stable rhizosphere interactions by sustaining carbon inputs to the microbiome.

In addition, the duration and intensity of drought strongly affect microbial dynamics. Short-term drought may induce reversible shifts in community composition, whereas prolonged or repeated stress can lead to long-term restructuring, reduced diversity, and the establishment of stress-adapted microbial assemblages. These factors collectively highlight that drought-induced microbiome responses are highly system-specific and must be interpreted within the context of soil properties, plant genotype, and stress history.

Functionally, drought selects microorganisms capable of maintaining metabolic activity under osmotic stress. These include taxa producing exopolysaccharides (EPS), osmolytes, and 1-aminocyclopropane-1-carboxylic acid (ACC) deaminase, which reduces stress-induced ethylene levels in plants (Das et al., 2025; Vurukonda et al., 2016). The reduction of ethylene levels by ACC deaminase-producing microbes helps prevent premature inhibition of root elongation, thereby promoting root growth and enhancing water uptake under drought conditions. Osmolyte and EPS production further contribute to maintaining cellular hydration and improving soil aggregation, which supports rhizosphere stability and water retention. These observations suggest that microbiome-mediated processes are not merely supportive but functionally integral to plant adaptation under drought stress, as they directly regulate key physiological responses such as root development, water uptake, and stress signaling.

Enhanced antioxidant activity and modulation of phytohormone biosynthesis are also closely linked to plant physiological responses, including the regulation of stomatal conductance, maintenance of photosynthetic activity, and protection against oxidative damage. Together, these processes help sustain root functionality, improve water-use efficiency, and stabilize plant metabolism under drought stress (Finkel et al., 2017; Siddique et al., 2023; Liu et al., 2025). In arid environments, drought-adapted microbial communities display specialized survival strategies that sustain nutrient cycling and stabilize rhizosphere interactions (Hanaka et al., 2021; Prisa and Jamal, 2025).

With climate projections indicating an increasing frequency and intensity of drought events, the risks to major crops, including rapeseed and sunflower, are expected to intensify (Zhao et al., 2025b). Therefore, integrating plant physiological responses with rhizosphere microbiome dynamics is essential for developing drought-resilient oilseed production systems.

Overall, drought does not merely suppress microbial diversity but restructures community composition and functional potential, favoring stress-adapted taxa that contribute to nutrient acquisition, oxidative stress mitigation, and hormonal regulation in oilseed crops.

The integration of physiological, molecular, and microbiological knowledge on drought will allow for the development of comprehensive plant protection strategies to increase the productivity of agricultural systems. Soil microorganisms thus demonstrate adaptive strategies to different types of abiotic stress, which identifies their key role in maintaining soil health and plant resilience to adverse conditions (**Table 2**). As summarized in **Table 2**, drought-induced shifts in microbial communities are consistently associated with the enrichment of stress-tolerant taxa and functional traits related to osmotic adjustment and antioxidant activity.

**Table 2:** Mechanisms of soil microbial influence on oilseed crop tolerance in abiotic stress.

The oilseed crops	Type of microorganism	Abiotic stress	Microbe influence	References
*B. napus* and *B. rapa* (canola)	*Pseudomonas, Pantoea, Azotobacter, Alcaligenes*	Drought, salinity, heavy metals	Phytohormone production; osmotic regulation; ACC deaminase activity; improved water uptake and photosynthesis	(Sridhar et al., 2025a; Ma et al., 2025)
*H. annuus*	*Pseudomonas stutzeri, Kushneria marisflavi, microbial consortia, AMF*	Drought, salinity, heavy metals	Antioxidant activation; ethylene regulation; osmolyte production; improved nutrient uptake and stress tolerance	(Gamalero et al., 2020; Bell et al., 2022; Purohit et al., 2024)
*Carthamus tinctoriu* (Safflower)	*Bacillus megaterium, B. subtilis*	Drought	Improved growth, yield, and seed quality; enhanced stress tolerance	(Jacoby and Kopriva, 2019)
*Arachis hypogaea* (Peanut)	*Rhizobacteriaceae*	Drought	Improved physiological and biochemical stress responses	(Bazany et al., 2022)
*Sesamum indicum* (Sesame)	Endophytes, AMF, PGPR (Azospirillum, Piriformospora, Bacillus)	Drought, salinity	Phytohormone regulation; osmotic balance; improved growth and productivity	(Sridhar et al., 2025a; Cordovez et al., 2019; Gamalero et al., 2020; Ramandi and Seifi, 2023)
*G. max* (Soybean)	*AMF, Bacillus, Pseudomonas, metal-resistant microbes*	Drought, heavy metals	Improved growth and nutrient uptake; reduced metal toxicity; antioxidant activation	(Tran et al., 2024; Wang et al., 2024a; Yuan et al., 2021.)
*Pisum sativum L.* (Pea)	*Rhizobium, PGPR (ACC deaminase)*	Nutrient deficiency, multiple stresses	Nitrogen fixation; phytohormone production; osmotic regulation; ethylene reduction	(Chaudhari et al., 2020; De Silva et al., 2025; Nadeem et al., 2019)
Other oilseed crops (*C. sativa, mustard (B.juncea*)).	*PGPR, N-fixers, AMF, endophytes*	Drought, salinity, temperature, heavy metals	Phytohormone production; osmotic regulation; nutrient mobilization; metal detoxification; antioxidant activity	(Petrić et al., 2022)

### 4.2 Salinity

Soil salinity, caused by the excessive accumulation of soluble salts such as sodium, calcium, magnesium chlorides and sulfates, represents a major abiotic constraint in oilseed production systems (Shrivastava and Kumar, 2015; Shokri et al., 2024). Approximately 20% of global arable land and 33% of irrigated areas are affected by salinization, resulting in yield losses ranging from 20–50% under climate change and unsustainable agricultural practices (Zhao et al., 2025b; Chele et al., 2021; Shokri et al., 2024). While salinity directly impairs plant physiology through osmotic stress, ion toxicity (Na^+^, Cl^-^), and nutrient imbalance (El-Ramady et al., 2024; Paul et al., 2019; Negacz et al., 2022), it simultaneously induces profound shifts in rhizosphere microbial communities.

Elevated soil conductivity reduces the activity of key enzymes involved in carbon and phosphorus cycling, including dehydrogenase and phosphatase, thereby limiting organic matter mineralization and nutrient turnover (Martins et al., 2023). These biochemical constraints alter microbial metabolic rates and reshape community composition. In general, salinity tends to suppress salt-sensitive taxa while selecting for halotolerant and halophilic microorganisms capable of maintaining cellular homeostasis under high osmotic pressure. For example, members of the general *Halomonas* and *Marinobacter* retain metabolic activity even at elevated salinity levels (Martins et al., 2023), reflecting adaptive traits such as compatible solute accumulation, ion transport regulation, and membrane stabilization.

Beyond their intrinsic stress tolerance, these microorganisms can contribute to plant resilience under saline conditions through multiple mechanisms. Halotolerant bacteria may enhance plant salt tolerance by producing osmoprotectants and exopolysaccharides that improve rhizosphere structure and water retention, as well as by regulating ion balance through selective uptake or sequestration of sodium ions. In addition, some strains are capable of modulating phytohormone levels and reducing stress-induced ethylene via ACC deaminase activity, thereby supporting root growth and maintaining physiological function under salinity stress.

Functionally, salinity-driven microbial restructuring influences nutrient bioavailability and rhizosphere interactions in oilseed crops (Ma et al., 2025). Changes in microbial enzyme activity affect carbon turnover and phosphorus mobilization, while salt-adapted plant growth–promoting rhizobacteria (PGPR) may mitigate stress through osmolyte production, phytohormone modulation, and regulation of ethylene levels.

These microbiome-mediated processes are closely linked to plant physiological and biochemical responses. For instance, microbial regulation of phytohormones contributes to the maintenance of root growth and architecture under saline conditions, while osmolyte production supports cellular osmotic balance and protects membrane integrity. In addition, microbial modulation of ion transport can reduce sodium accumulation and improve potassium retention, thereby maintaining ion homeostasis and preventing toxicity. Enhanced antioxidant activity induced by associated microbiota helps limit reactive oxygen species (ROS) damage, supporting photosynthetic efficiency and overall metabolic stability.

Collectively, these interactions support plant water uptake, regulate stomatal conductance, and contribute to sustained growth under saline stress. However, combined stress conditions, such as salinity and drought, may intensify physiological constraints and reduce overall plant performance.

Overall, salinity does not merely act as a plant-level stressor but functions as a selective ecological filter within the rhizosphere, reshaping microbial diversity, metabolic activity, and nutrient cycling processes. These microbial adjustments are critical determinants of soil health and indirectly influence oilseed productivity under saline environments.

### 4.3 Temperature impact

Extreme temperatures, including both heat and cold stress, represent major abiotic constraints that reduce oilseed productivity worldwide (Khan et al., 2025a; Sapakhova et al., 2023). While high and low temperatures directly impair plant physiological processes, such as photosynthesis, membrane stability, enzymatic activity, and reproductive development (Wang et al., 2019; Knight et al., 2024; Li et al., 2024; Zhang et al., 2024a), they also exert strong selective pressure on soil microbial communities, particularly within the rhizosphere.

Heat stress alters soil moisture dynamics, accelerates organic matter turnover, and modifies root exudation patterns, thereby reshaping microbial community structure. Elevated temperatures may reduce overall microbial diversity and network complexity, while favoring thermotolerant taxa capable of maintaining membrane stability and enzymatic function under thermal stress. Such shifts can influence carbon and nitrogen cycling rates and modify nutrient availability to oilseed crops. At the plant level, heat stress induces protein denaturation, membrane destabilization, impaired photosynthesis, pollen sterility, and premature senescence, ultimately reducing yield (Wang et al., 2019; Knight et al., 2024). These physiological disturbances indirectly affect rhizosphere microbial interactions by altering the quantity and composition of root-derived substrates.

Cold stress similarly restructures microbial communities through reduced metabolic activity, slower decomposition rates, and altered redox conditions. In plants, low temperatures cause membrane damage, reactive oxygen species (ROS) accumulation, and suppression of gene expression and growth processes (Li et al., 2024; Zhang et al., 2024a). Prolonged exposure can lead to reduced germination, developmental delays, and even early plant mortality (Li et al., 2024). From a microbiological perspective, low temperatures select for psychrotolerant or cold-adapted microorganisms capable of maintaining enzymatic activity under reduced thermal conditions. These microbes contribute to sustaining nutrient cycling and stabilizing rhizosphere interactions despite constrained metabolic rates.

Overall, temperature extremes function as ecological filters that reshape microbial diversity, metabolic activity, and functional gene expression in oilseed cropping systems. The resulting microbial restructuring influences nutrient turnover, oxidative stress mitigation, and rhizosphere stability, thereby indirectly modulating plant resilience under fluctuating thermal environments.

### 4.4 Heavy metal

Heavy metal (HM) contamination of soils is one of the most stable manifestations of anthropogenic pressure on agroecosystems. Elements such as cadmium (Cd), lead (Pb), zinc (Zn), nickel (Ni), copper (Cu), and molybdenum (Mo) disrupt the structural and functional organization of soil microbial communities, reducing diversity, metabolic activity, and symbiotic potential (Iimaa et al., 2025; Khoso et al., 2024; Daurov et al., 2024). Heavy metals interact with cell membrane and enzyme components, inducing oxidative stress, DNA damage, and reduced respiratory activity of microorganisms. Oil crops, including *B. napus*, *H. annuus*, and *C. sativa*, are sensitive to the accumulation of heavy metals in the rhizosphere. However, certain rhizobacteria and arbuscular mycorrhizal fungi (AMF) mitigate the toxicity of metals by binding them to inactive forms, enhancing antioxidant activity, and activating the expression of stress response genes (Patyal et al., 2025). For example, PGPR, which produce siderophores, reduces the uptake of Cd and Pb in B. napus tissue and increases the activity of catalase (CAT), superoxide dismutase (SOD), and peroxidase (POD) enzymes (Iimaa et al., 2025; Ansabayeva et al., 2025). Metagenomic studies show that heavy metal resistant microbial communities are enriched with arsR, copA, czcA, and merA genes associated with detoxification of metal ions (Iimaa et al., 2025; Wu and Bose, 2024). The presence of AMF enhances plant stabilization of Cd and Zn by sequestration of metals in hyphae and enhancement of root barrier function (Patyal et al., 2025). Thus, soil microbiology plays a key role in buffering the toxic effects of heavy metals and increasing the resilience of oilseeds. The combined use of PGPR and AMF is seen as a promising biotechnological strategy for remediation of contaminated agroecosystems (Ansabayeva et al., 2025; Sahoo et al., 2024).

Stress conditions tend to reduce microbial diversity, while promoting the development of stress-tolerant taxa capable of supporting key biogeochemical processes under adverse conditions. These changes in microbial communities are crucial for soil health and indirectly affect the productivity of oilseeds under changing climate conditions (Patyal et al., 2025; Wang et al., 2025b).

Overall, abiotic stress significantly reshapes the structure, diversity, and functional activity of soil microbial communities in oilseed cropping systems, thereby influencing soil health and indirectly affecting crop performance under adverse environmental conditions.

## 5 A role of microbiome in the response of oilseed crops to abiotic stress

### 5.1 Microbial regulation of drought and salinity tolerance

Abiotic stresses substantially alter rhizosphere microbial composition and function (Section 3), yet soil microbiota also plays an active role in enhancing plant adaptation. In oilseed crops (particularly *Brassica* spp.), stress-adapted microorganisms such as plant growth-promoting rhizobacteria (PGPR) and arbuscular mycorrhizal fungi (AMF) mediate tolerance to drought, salinity, extreme temperatures, and heavy metal toxicity through multiple coordinated mechanisms (Parastesh et al., 2025). Compared to many cereal crops, oilseeds often exhibit a stronger dependence on AMF for phosphorus acquisition and osmotic adjustment under combined drought–salinity stress, while PGPR strains more consistently promote ABA-mediated stomatal regulation and ROS scavenging in *B.napus* and *B. juncea* under drought. These taxa improve root hydraulic conductivity, maintain higher relative water content, enhance antioxidant enzyme activities (e.g., CAT, POD, SOD), modulate phytohormone balance (IAA, cytokinins, ABA), and upregulate stress-responsive genes (e.g., *WRKY*, *MYB*, *LEA*) to a greater extent in tolerant doubled-haploid oilseed lines than in sensitive parental genotypes. Such oilseed-specific responses suggest that microbiome-based strategies may offer particularly high potential for improving resilience in *Brassica* oilseed systems under multiple abiotic constraints. Although abiotic stress alters soil microbial composition (Section 3), microorganisms actively contribute to oilseed stress adaptation through multiple physiological and molecular pathways. In rapeseed, PGPR-mediated phytohormone regulation and antioxidant activation are relatively well documented, whereas in camelina such mechanisms remain insufficiently explored. AMF associations have demonstrated improved phosphorus uptake and drought tolerance in sunflower systems (Parastesh et al., 2025), yet comparative multi-species analyses are still lacking.

Studies with canola (*B. napus*) have shown that the combined use of PGPR and biochar in water deficient conditions increases relative leaf moisture and the activity of antioxidant enzymes such as POD, SOD, and glutathione reductase (GR), as well as the concentration of macronutrients (N, P, K) compared to plants exposed only to drought (Singh et al., 2020). Metabolomic analysis of *B. juncea* after PGPR treatment revealed increased synthesis of osmoprotectants, amino acids, and sugars that support membrane stability and repair damage caused by water deficiency (Singh et al., 2020). Under salinity conditions, PGPR inoculants that produce biofilms and ACC-deaminase contribute to increased activity of the antioxidant enzymes CAT, SOD, and glutathione peroxidase (GPX) in sunflowers, reducing oxidative stress.

Hormonal signaling pathways also play a key role - phytohormones such as abscisic acid (ABA), IAA, and cytokinin regulate the processes of stomatal closure, root growth, transpiration, and stress signal adaptation. Studies on rapeseed indicate that, in drought, long non-coding RNAs (lnc-RNAs) coordinate the pathways of transmission of phytohormonal signals, influencing the expression of genes responsible for stress. When salting, the combined action of AMF and PGPR promotes membrane integrity, improves mineralization, and maintains osmotic potential, reducing water loss and toxic ion levels (Cheng et al., 2022).

A critical component of stress adaptation is the reinforcement of antioxidant defense through enzymes such as superoxide dismutase, catalase, and peroxidases, which neutralize excessive ROS generated during environmental stress. Regulation of heat shock proteins (HSPs), dehydrins, and ion transporters further contributes to maintaining structural integrity and metabolic stability under adverse conditions (Bhat et al., 2023).

### 5.2 Microbial mechanisms of heavy metal detoxification and phytoremediation

In addition to adaptation to drought and salt stress, microbiology is actively involved in neutralizing the toxicity of heavy metals. Thus, PGPR can bind and inactivate heavy elements through biosorption, extracellular capsule formation, intracellular chelation, and metabolic transformation (Sahoo et al., 2025; Liu et al., 2024). These mechanisms are used in phytoremediation: microorganisms either reduce the accumulation of metals in plants (phytostabilization) or enhance their extraction (phytoextraction), depending on the purpose of ecological restoration (Asad et al., 2019).

Endophytic microorganisms located inside plant tissues play a key role in regulating physiological processes: they stimulate growth through the synthesis of phytohormones (e.g., IQC, cytokines, ABK, and gibberellin) and osmoprotectors and pro-duce ACC-deaminase. Some bacterial strains of the genera *Rhizobium*, *Pseudomonas*, *Stenotrophomonas*, and *Rhodococcus* are also involved in organic pollutant decomposition, contributing to complex soil remediation (Franco-Franklin et al., 2021).

Thus, soil microbiology plays a crucial role in the adaptation of oilseeds to abiotic stresses - drought, salting, and heavy metal pollution. Favorable microorganisms activate the antioxidant system of plants, regulate water and ion balance, enhance photosynthesis, and stabilize cell membranes (Serero et al., 2021). The introduction of microbial inoculants, such as *Azospirillum brasilense* and *Bacillus circulants*, has already demonstrated effectiveness in increasing cereal crop yields under stress conditions, opening wide opportunities for similar approaches in oilseed farming (Jakhar et al., 2020).

### 5.3 Coevolutionary relationships and climate-driven microbiome dynamics

The ecological relationships between oilseeds and soil microbiota form an extensive system of mutual adaptation developed over a long period of coevolution. In such a system, plants function not as autonomous organisms but as part of microbial networks providing trophic, signaling and protective functions (Jakhar et al., 2020; Liu et al., 2021a). Oilseeds, such as *B. napus*, *C. sativa* and *H. annuus*, establish stable associations with rhizosphere bacteria, arbuscular mycorrhizal fungi (AMF), *Actinobacteria* and *Pseudomonas*, which improve nutrient availability and increase resistance to abiotic stress (Verbon and Liberman, 2016).

The coevolution of plants and microbes has led to the formation of mutual signaling pathways regulating symbiosis: roots secrete a wide range of compounds - sugars, amino acids, phenolic and organic acids - acting as substrates channeling for microorganisms (Liu et al., 2021a; Jiménez et al., 2020). In response, the microbes activate the expression of genes responsible for the colonization of the rhizosphere, the formation of biofilms, and the synthesis of phytohormones (IAA, GA, ABA) (Khoso et al., 2024). Notably, microalgae also contribute to phytohormone production; genetic evidence demonstrates IAA biosynthesis in the model alga *Chlamydomonas* reinhardtii via an extracellular L-amino acid oxidase (*LAO1*)-mediated pathway from L-tryptophan, particularly under nitrogen limitation, where IAA mediates chemical crosstalk and supports mutualistic interactions with bacteria (e.g., *Methylobacterium* spp.) by modulating algal growth inhibition and bacterial auxin degradation (Arenas et al., 2025; Liu et al., 2021b; Khoso et al., 2024). Although primarily studied in aquatic or phycosphere contexts, such mechanisms suggest broader implications for rhizosphere-like environments where microalgae may co-occur and influence phytohormone pools available to plants.

For the family *Brassicaceae*, which includes oilseeds like *B. napus*, especially important role played by bacterial associations of roots (eg, *Pseudomonas*, *Bacillus*, *Rhizobium, Arthrobacter*). They increase stress resistance by regulating the hormonal balance and activating the antioxidant system of plants. For example, PGPR strains isolated from *B. napus* increased the proline concentration and activity of SOD, CAT and POD enzymes in drought (Dobrzyński et al., 2025). At the same time, mycorrhizal fungi help to improve phosphorous nutrition and heavy metal sequestration in other oilseeds such as *H. annuus* (Vangelisti et al., 2019).

### 5.4 Climate-driven microbiome shifts and agroecosystem resilience

These crop-specific microbial partnerships respond dynamically to climatic stressors. Temperature extremes and moisture fluctuations drive seasonal microbiome shifts: drought favors spore-formers (*Bacillus, Clostridium*) while excess moisture enriches *Pseudomonas* and *Burkholderia denitrifiers* (Verbon and Liberman, 2016). Rapeseed and sunflower studies confirm stress-adaptive transitions in functional guilds, particularly carbon metabolism and antioxidant pathways (Jiménez et al., 2020). Oilseed rhizospheres thus serve as “stress sentinels” with *B. napus* recruiting more osmolyte producing *Bacillus* under drought, while sunflower favors salinity-tolerant *Pseudomonas* for ROS scavenging (Zverev et al., 2023). Beyond direct plant benefits, these interactions regulate whole agroecosystem resilience. Microbes mediate nutrient cycling, carbon sequestration, and trophic cascades, while oilseed root exudates (phenolics, sugars) shape microbial recruitment, sustaining long-term soil fertility (Blakney et al., 2023). Comparative analysis reveals oilseed-specific patterns: rapeseed exudes more flavonoids favoring PGPR, sunflower prioritizes organic acids for AMF, creating genotype-tailored microbiomes that buffer multi-stressor impacts (Chen et al., 2024).

Microbiome-mediated mechanisms, such as phytohormonal balance, antioxidant activation, osmotic adjustment, and metal detoxification can enhance oilseed productivity under abiotic stress. For example, *B. napus* has been associated with improved drought tolerance linked to auxin-mediated responses, *H. annuus* with mechanisms related to salinity exclusion and ion regulation, and *C. sativa* with enhanced heavy metal tolerance and phytoextraction capacity, including pathways involving glutathione metabolism (Chen et al., 2024).

These complementary strategies optimize agroecosystem resilience under intensifying climate pressures. Climatic factors, especially temperature and humidity, regulate the seasonal dynamics of microbiology. When there’s drought, the proportion of bacteria that form spores (*Bacillus* and *Penicillus*) rises, and when humidity rises, *Pseudomonas* and *Burkholderia* are seen (Blakney et al., 2023). Studies on rapeseed and sunflower showed that the microbial community can adapt to stress through changes in functional groups associated with carbon metabolism and antioxidant activity (Zverev et al., 2023).

**Figure 3:**
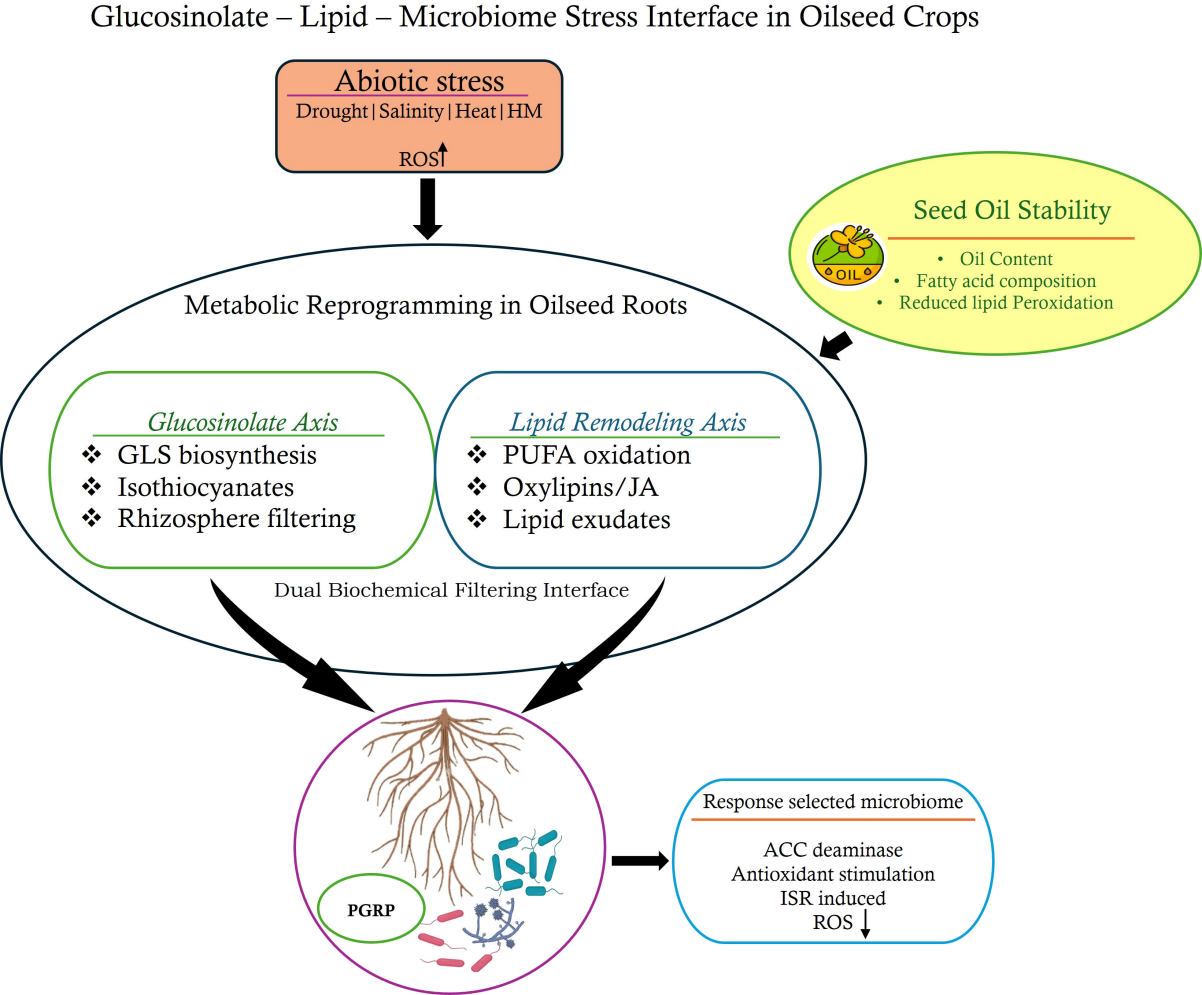
Schematic representation of the glucosinolate–lipid–microbiome stress interface in oilseed crops (primarily *Brassicaceae*). Abiotic stresses trigger reactive oxygen species (ROS) accumulation, inducing metabolic reprogramming in roots via the glucosinolate axis (GLS biosynthesis, isothiocyanate release, rhizosphere filtering) and lipid remodeling axis (PUFA oxidation, oxylipin/JA signaling, lipid exudates). This dual biochemical interface selectively assembles a stress-responsive rhizosphere microbiome, which in turn enhances antioxidant defenses, induces ISR, reduces ROS, and stabilizes seed oil content, fatty acid composition, and lipid peroxidation levels.

Although no microbiome−mediated process is strictly unique to oilseed crops, species within the *Brassicaceae* family display a distinctive stress−induced rhizosphere filtration mechanism that integrates glucosinolate production with lipid signaling, shaping selective microbial assemblages under adverse conditions. Glucosinolates are sulfur− and nitrogen−containing secondary metabolites that accumulate in *Brassica* roots under abiotic stress and act as chemical signals influencing belowground microbial recruitment and plant microbe feedback cycles, with evidence of reciprocal effects between glucosinolate profiles and rhizosphere community structure in *B. rapa* (Muthusamy and Lee, 2024) (**Figure 3**).

Root exudates, including specialized metabolites, are key drivers of rhizosphere dynamics and microbial assembly, facilitating beneficial interactions that influence hormonal and defense signaling networks under stress (Ren et al., 2026). These microbiome feedback cycles enhance plant antioxidant defenses and contribute to the stabilization of seed oil biosynthesis and lipid quality, supporting stress resilience in *Brassicaceae* oilseeds in comparison to non−oilseed cereals.

Plant - microbial interactions go beyond individual effects and shape the resilience of the entire agroecosystem. The microbe regulates trophic linkages, element circulation and carbon balance, and the oilseeds through root secretions create favorable conditions for the microbe, maintaining soil fertility (Chen et al., 2024). Microbiome-mediated processes, such as phytohormonal regulation, antioxidant defense activation, osmotic adjustment, and metal detoxification, can have a significant impact on enhancing abiotic stress tolerance and productivity of oilseed crops (Venter et al., 2016).

## 6 Multi-omics-driven microbiome biotechnologies for abiotic stress tolerance in oilseed crops

**Figure 4:**
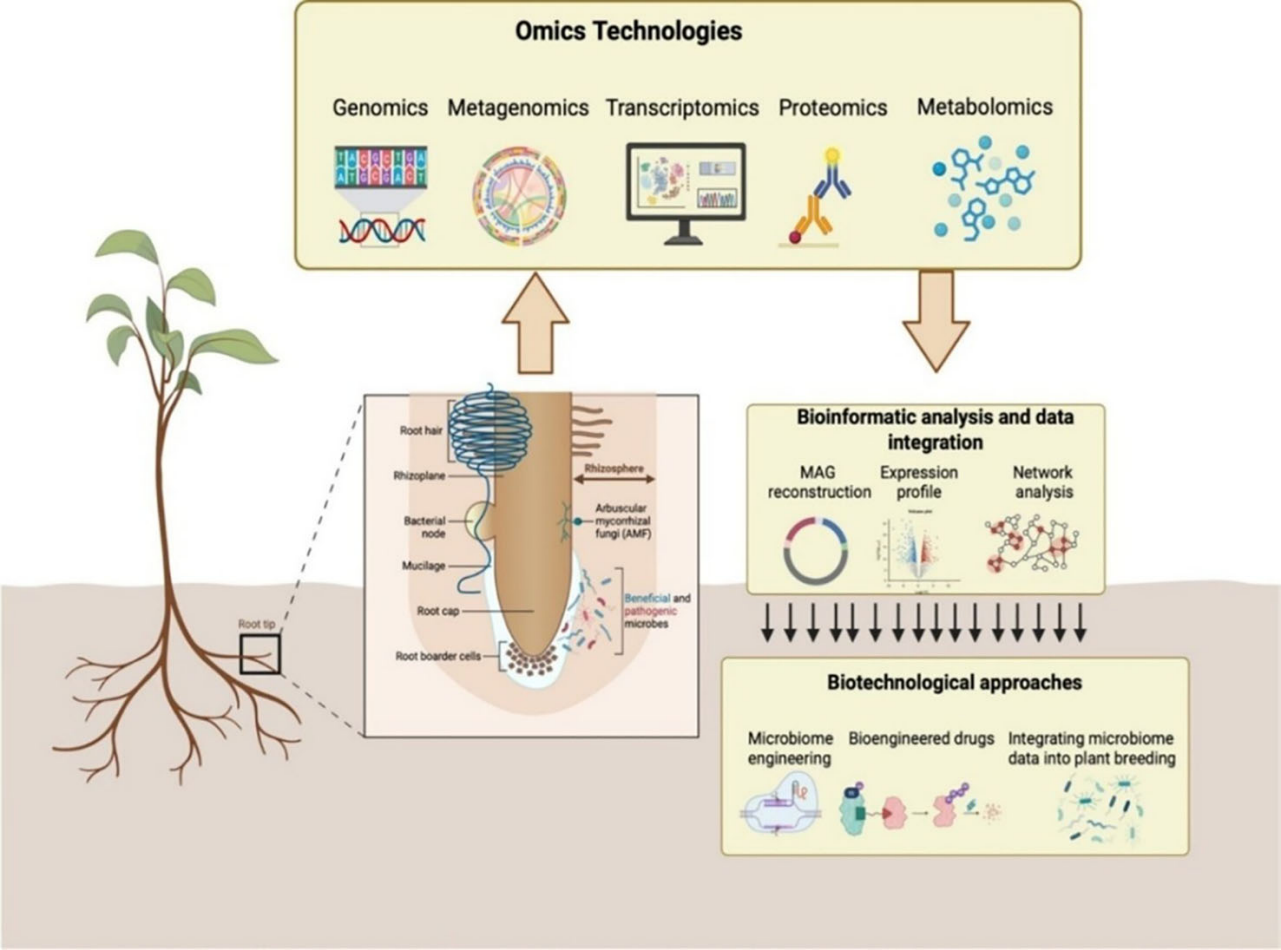
Schematic overview of multi-omics technologies used to study plant roots-related microbiology, their bioinformatics integration and biotechnology applications (created by BioRender).

In recent years, rapid advances in omics technologies, genomics, transcriptomics, proteomics, metabolomics, and metagenomics have fundamentally reshaped how we study microbial communities and their interactions with plants (**Figure 4**). **Figure 4** provides an overview of multi-omics approaches and their integration, highlighting how different data layers contribute to understanding microbiome function and plant-microbe interactions. To fully capture microbiome functionality, these approaches must be integrated within a unified analytical framework, as each omics layer provides complementary insights ranging from taxonomic composition and genetic potential to gene expression, protein activity, and metabolite production.

Multi-omics technologies combined with bioinformatic integration reveal key plant-microbe interactions and support microbiome-based biotechnological applications and climate-resilient crop improvement. For instance, metagenomics can identify stress-responsive microbial taxa and genes, while metatranscriptomics and proteomics reveal their active expression under specific environmental conditions. Metabolomics further links these processes to biochemical outputs that directly influence plant physiology, including osmolytes, phytohormones, and signaling compounds.

These powerful approaches allow us to see the microbiome not as a simple assembly of individual microbes, but as a highly integrated functional network whose metabolic pathways are closely linked to plant physiology and respond dynamically to abiotic stresses. Such integrative analyses enable the identification of key functional pathways and microbial consortia involved in stress adaptation, providing a mechanistic and systems-level understanding of plant microbiome interactions.

### 6.1 Microbial inoculants and synthetic communities

Modern soil microbiology studies are opening new perspectives for biotechnology in agriculture, aimed at increasing the resistance of plants to abiotic stresses. Rhizosphere microorganisms - including rhizobacteria, cyanobacteria, actinomycetes and arbuscular mycorrhizal fungi (AMF) - perform key functions in plant nutrition, protection and growth regulation (Abdul Rahman et al., 2021). In conditions of abiotic stress such as drought, salinity and accumulation of heavy metals, the activation of symbiotic interactions between plants and microorganisms plays a key role in reducing stress and maintaining physiological activity of crops. Rhizosphere microbial communities can modulate antioxidant status, water balance and nutrient uptake, which is especially important for oilseed crops including *B. napus*, *H. annuus* and *C. sativa* (Gentsch et al., 2020).

One of the promising areas is the development and use of microbial inoculants, preparations containing live cells of beneficial microorganisms (PGPR, AMF), capable of stimulating plant growth and increasing their resistance to stress. Thus, representatives of PGPR (plant-growth-promoting rhizobacteria) realize their functions through the production of phytohormones (auxins, cytokines, gibberellins), synthesis of siderophores, nitrogen fixation, dissolution of phosphates and induction of systemic stability (ISR) (Akimbekov et al., 2020; Compant et al., 2019; Adeleke et al., 2024). AMF interaction with the root system enhances nutrient uptake, especially of phosphorus and micronutrients, as well as reduces oxidative stress by activating antioxidant enzymes (Adeleke et al., 2024). For oilseeds, microbial inoculants demonstrated significant increases in drought and salinity resistance. For example, in the treatment of *B. napus* seeds by PGPR consortium (strains of *Bacillus subtilis* and *Pseudomonas fluorescens*) increased activity of catalase (CAT), peroxidase (POD) and superoxide dismutase (SOD), which led to reduced accumulation of active oxygen forms, such mechanisms are supported by general reviews of the role of PGPR in drought and salt stress (Kibret et al., 2024).

Traditional inoculants based on individual strains are inferior in efficiency to complex microbial consortia, where microorganisms with different physiological functions are combined. Such synthetic communities (SynComs) are formed based on targeted selection of strains that interact synergistically: PGPR activates the plant’s hormonal response, and AMF enhances trophic functions and metabolic resistance (Ullah et al., 2025). Synthetic microbial consortia (SynComs) are emerging as a promising approach for enhancing plant stress tolerance, although their effectiveness under field conditions remains to be fully validated. For example, microbial consortia combining plant growth–promoting bacteria and mycorrhizal fungi have been reported to enhance plant performance under water deficit conditions through mechanisms such as improved osmotic regulation and stress-responsive gene expression. However, the effectiveness of specific combinations remains highly context-dependent and requires further validation under field conditions (Chouhan et al., 2021).

Forming such systems require a deep understanding of microbial interactions and signaling pathways. Modern metagenomics techniques enable the identification of key taxa and functional genes such as those involved in the synthesis of triglycerides, glycerophospholipids and exopolysaccharides that provide microbial communities with resistance to desiccation and other stress factors (Afridi et al., 2022; Anthony et al., 2024).

Field inconsistency is often associated with variability in soil properties, including pH, physicochemical conditions, and the composition of native microbial communities, which can substantially influence the efficacy of microbial inoculants. In addition, reduced survival and establishment of introduced microorganisms under drought and salinity conditions further contribute to inconsistent outcomes (Cerecetto et al., 2021). SynComs show promise in lab/greenhouse trials but falter in diverse field soils due to competitive exclusion by residents (Li et al., 2023). Regulatory hurdles, such as rigorous EPA/EU approvals requiring multi-year stability data, delay commercialization and limit farmer access, especially in low-resource regions. Costly scale-up and lack of standardized formulations exacerbate adoption barriers. Overcoming these demands soil-tailored SynComs, advanced delivery (e.g., nano-encapsulation), and policy reforms for faster registration (Hidri et al., 2025).

Despite the promising potential of microbiome-based strategies, their large-scale implementation in agricultural systems remains challenging. Field-level performance often differs from controlled laboratory or greenhouse conditions due to environmental variability, including soil heterogeneity, climate fluctuations, and interactions with native microbial communities. These factors can influence the establishment, persistence, and functional stability of introduced microbial inoculants, frequently leading to inconsistent outcomes across locations and growing seasons.

Scalability also presents a significant constraint, as the production, formulation, and delivery of effective microbial consortia require optimization to ensure viability and performance under diverse field conditions. In addition, adoption by farmers depends on factors such as cost-effectiveness, ease of application, regulatory approval, and demonstrated yield benefits under real-world conditions.

Addressing these challenges will require long-term field trials, site-specific microbiome management strategies, and the integration of agronomic practices with microbial technologies. Bridging the gap between laboratory findings and field application remains a critical step toward the successful deployment of microbiome-based solutions for sustainable oilseed production under abiotic stress.

### 6.2 Multi-omics technologies in microbiome research

Genomic and metagenomic studies have unlocked the genetic potential of soil microbiota, including uncultivated species, enabling the identification of genes involved in tolerance to salinity, drought, and heavy metal toxicity (Cerecetto et al., 2021; Lau et al., 2022). High-throughput next-generation sequencing (NGS) and metagenomics have dramatically expanded our understanding by revealing the vast taxonomic and functional diversity of rhizosphere microbes in oilseed crops, including many previously unculturable taxa that traditional culturing methods overlooked (Ali et al., 2022; Anzano et al., 2022). Metagenomic analyses have uncovered novel gene families and functional pathways in these communities, such as those involved in osmotic regulation (e.g., proline biosynthesis-related genes), antioxidant defense (e.g., catalase, superoxide dismutase, and peroxidase sequences), ion transport for salinity tolerance (e.g., NHX sodium/hydrogen antiporter and HKT high-affinity potassium transporter families), sulfur assimilation (e.g., SULTR sulfate transporter variants), and metal detoxification (e.g., metallothionein-like, glutathione-related, phytochelatin synthesis, and GGCT gamma-glutamyl cyclotransferase pathways), directly linking microbial functions to plant stress adaptation. These studies have established correlations between microbiota composition and plant tolerance to drought, salinity, and other types of abiotic stress (Isgandarov et al., 2026). For instance, in *B. napus* rhizospheres, metagenomics has shown stage-dependent shifts in microbial composition, with enrichment of stress-protective taxa during growth phases that influence phytohormone signaling and phytoprotection (Wang et al., 2024b). Domestication studies further reveal reduced microbial diversity in cultivated forms compared to wild relatives, highlighting how breeding has altered beneficial associations (Zhang et al., 2023).

It has also been established that the structure of the *B. napus* seed microbiome depends on the variety and determines the interaction of symbionts and pathogens (Rybakova et al., 2017). In *C. sativa*, core microbial communities maintain functional stability under varying moisture, while specific taxa support metabolic adjustments to stress through enhanced nutrient cycling and protective mechanisms, including glutathione-mediated pathways (Barnes et al., 2025; Neupane et al., 2022). Modern metagenomic studies have revealed promising novel approaches for investigating existing microorganisms, while identifying new microbial strains and their genes that actively enhance oilseed crops’ resilience to drought, salinity, and heavy metals (Yao et al., 2026; Isgandarov et al., 2026).

### 6.3 Bioinformatics integration and machine learning

Combining multi-omics technologies with machine learning and artificial intelligence enables predictive microbiome modeling, supporting the design of personalized biopreparations for specific varieties, soil types, and stress conditions, while optimizing agricultural practices and reducing chemical inputs. In *B. napus* (rapeseed/canola), machine learning (ML) models integrating rhizosphere microbiome features (from amplicon sequencing) with phenotypic imaging data predict yield and yield stability early (e.g., third week post-planting), achieving high accuracy (R² 0.81–0.91) by identifying key taxa linked to nutrient mobilization and stress protection across phenological stages (Aziz, 2022). ML algorithms (e.g., random forest) applied to environmental and agronomic data in canola further enable precise nitrogen recommendations and yield forecasting, indirectly incorporating microbiome dynamics for nutrient efficiency under abiotic stress (Ma et al., 2025). In saline-alkaline contexts relevant to oilseeds, ML analyzes soil parameters to predict optimal PGPR consortia (e.g., *Bacillus*–*Pseudomonas*) for microbiome synergy and enhanced remediation/yield (Wen et al., 2021). These examples illustrate practical translation to tailored bioinoculants and sustainable management in oilseed crops.

### 6.4 Microbiome engineering and biopreparations

The field of microbial engineering sits at the intersection of systems biology, genome editing, and bioinformatics, aiming to purposefully design microbial communities with targeted beneficial traits. This involves diverse methods: metagenomic soil analysis identifies promising microbes and genes, while bioengineering approaches enable their enhancement or modification (Thakur et al., 2023). CRISPR/Cas technologies play a particularly vital role, actively editing genes responsible for microbial stress tolerances such as those encoding antioxidant enzymes, transport proteins, and signaling pathway regulators (Ding et al., 2020; Sapakhova et al., 2025). The result is the creation of “smart” microbes capable not only of surviving extreme environments but also of positively influencing plant growth (Dudeja et al., 2025; Ramachandran and Bikard, 2019).

New generation biopreparations based on encapsulated microbial cells, bacterial cells and carriers are also being actively developed to ensure stability and controlled release of active microorganisms in the soil. Selecting stress-tolerant microbial strains and consortia, particularly PGPR and AMF, plays a pivotal role in enhancing the survival and productivity of oilseed crops under extreme conditions (Hidri et al., 2025). These microorganisms establish a resilient rhizosphere capable of adapting to fluctuations in moisture and temperature. Integrating advanced methods like microbial engineering, synthetic microbial communities, and agroecological practices (minimal soil tillage, cover crops, herbicide application, and organic fertilizers) forms the foundation for sustainable agriculture (Dubey et al., 2025). This approach shifts from isolated microorganisms to complex, coevolved microbial communities (Dubey et al., 2025).

Thus, developing stress-tolerant strains and consortia, combined with agroecological practices and SynComs, offers a promising systems strategy for improving oilseed resilience, productivity, and long-term viability in harsh environments (Ramachandran and Bikard, 2019; Li et al., 2023b; Yang et al., 2025a). Despite their potential, microbial consortia and bioinoculants face critical limitations in practical deployment. **Table 3** presents the main metagenomic and biotechnological approaches aimed at managing soil microbiome and increasing crop resistance to abiotic stresses.

**Table 3:** Basic metagenomic and biotechnological approaches aimed at managing soil microbiome and increasing crop resistance to abiotic stresses.

Approach	Biotechnological approach	Function	Examples of application in oilseeds	References
Metagenomics + metatranscriptomics	DNA/RNA profiling of microbial communities	Identification of stress-responsive genes and pathways	Detection of stress-adapted Pseudomonas and Bacillus in B. napus	(Xing et al., 2023)
Functional metagenomics	Gene screening and expression analysis	Discovery of beneficial traits (e.g., antioxidant activity, osmolyte production)	Development of stress-tolerant Rhizobium and Azospirillum strains	(Nwachukwu and Babalola, 2022)
Synthetic microbial consortia (SynComs)	Combination of functional microbes (PGPR, AMF, endophytes)	Synergistic enhancement of nutrient uptake and stress tolerance	*Bacillus + Pseudomonas* consortia for rapeseed	(El-Saadony et al., 2024)
Microbiome engineering	Targeted modification of microbial communities	Optimization of plant microbiome interactions	Engineering PGPR for enhanced phytohormone production	(Joshi et al., 2025)
Microbiome-assisted breeding	Integration of microbiome data into crop selection	Selection of stable and stress-resilient genotypes	Selection of drought-adapted *B. napus* varieties	(El-Saadony et al., 2024; Fadiji et al., 2023)
Bioinoculants and formulations	Development of microbial-based products	Improved inoculant stability and field performance	Bacillus-based formulations for sunflower and safflower	(Francioli et al., 2025)
Omics integration and modeling	Multi-omics data integration and predictive modeling	Prediction of microbiome behavior under stress	Modeling C. sativa–microbiome interactions under drought	(Radzali et al., 2025)

Thus, the integration of omics technologies and biology systems is becoming the foundation for the transition to intelligent microbiome management, where decisions are made based on comprehensive data analysis - from the genome to the agroecosystem level. Combining the data of metagenomics, proteomics and metabolomics makes it possible to create microbial compositions optimized for specific types of soils and crops (Clagnan et al., 2024). The use of this approach can lead to significant increases in productivity and sustainability for oilseed crops grown in arid regions, with minimal environmental impact.

## 7 Agronomic drivers of soil microbiome functioning and abiotic stress tolerance in oilseed crops

### 7.1 Agronomic practices and soil microbiome dynamics

Agronomic practices constitute key anthropogenic drivers that directly and indirectly modulate soil physicochemical properties and biological processes. Fertilization regimes, organic amendments, tillage intensity, and crop residue management profoundly influence soil pH, nutrient availability, salinity, and organic carbon content. These factors collectively define the abiotic environment for soil microbial communities, thereby shaping microbiome composition, diversity, and functional potential. As controllable variables, agronomic practices represent critical levers for enhancing microbiome-mediated resilience in oilseed crops facing intensifying abiotic stresses.

Agronomic interventions serve as primary determinants of soil and plant-associated microbiome structure and functional capacity, with cascading effects on crop productivity and stress tolerance. Intensive mechanical disturbances—such as plowing, harrowing, and deep inversion—alter soil aeration, organic matter distribution, and nutrient cycling, thereby reshaping microbial habitats, diversity, and metabolic activities (Dong et al., 2024). Shifts in fertilization and cropping systems further modulate key microbial guilds involved in nutrient transformations, particularly nitrogen cycling, which governs plant nutrient acquisition and growth. Moreover, practices like crop rotation and cover cropping restructure rhizosphere communities by modifying root exudation patterns and soil biochemical profiles (Seitz et al., 2024; Town et al., 2023).

**Figure 5:**
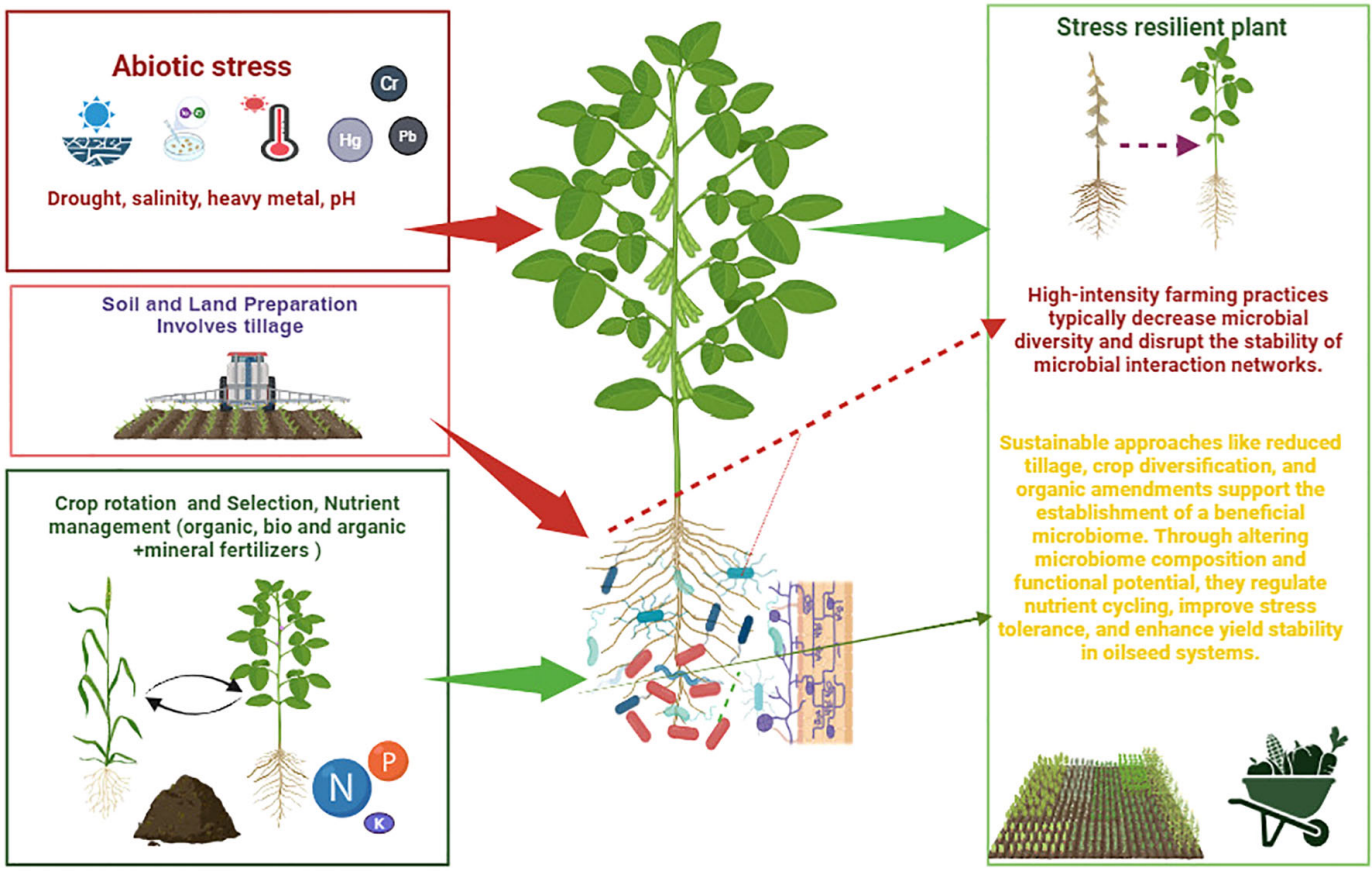
Conceptual schematic of agronomic practices modulating soil microbiome dynamics and abiotic stress resilience in oilseed crops (created by BioRender).

In oilseed crops such as *B. napus*, agronomic management interacts strongly with the rhizosphere microbiome to influence performance under nutrient limitation and abiotic stress. Such interventions alter root metabolite profiles and soil nutrient dynamics, selectively enriching beneficial taxa that mobilize nutrients and produce phytohormones (Lidbury et al., 2022). These microbes enhance nitrogen and phosphorus mineralization, promote plant growth, and confer tolerance to stresses including nutrient deficiency and drought (Wang et al., 2024d). Legume-inclusive rotations, for instance, reshape rhizosphere bacterial communities, favoring taxa such as Sphingomonadaceae and thereby boosting growth and yield in subsequent oilseed rape crops (Wang et al., 2024d) (**Figure 5**).

Intensive agronomic practices often reduce microbial diversity and destabilize interaction networks, whereas sustainable approaches, such as reduced tillage, diversified rotations, and organic amendments, foster beneficial microbiome assembly. By modulating microbiome composition and functional potential, these practices regulate nutrient cycling, plant stress tolerance, and yield stability in oilseed systems.

### 7.2 Mineral, organic, and integrated fertilization strategies and their microbial impacts

Long-term mineral fertilizer application markedly alters soil microbial communities. Reviews of extended experiments indicate that continuous mineral inputs often decrease diversity and shift composition toward bacterial dominance, frequently at the expense of fungi and symbiotic taxa (Kurzemann et al., 2020). Beyond nitrogen, phosphorus and potassium fertilizers influence microbiome structure and function. Elevated phosphorus availability diminishes plant reliance on phosphate-solubilizing bacteria and arbuscular mycorrhizal fungi, reducing their abundance (Zhong et al., 2010). Such shifts impair nutrient uptake and stress tolerance, particularly under drought or reduced fertilizer regimes. Thus, while mineral fertilizers ensure short-term nutrient supply, prolonged use may undermine microbial diversity and functional stability.

Under abiotic stress, soil microbial biomass (SMB) in rapeseed (*B. napus*) declines significantly, often by 30–50% under conditions such as drought or combined stresses, which impedes nitrogen mineralization processes and contributes to reduced seed oil content (Raza et al., 2021; Zhu et al., 2023). Similarly, salinity stress suppresses sunflower SMB and associated phosphatase activity, thereby constraining phosphorus uptake and leading to substantial yield reductions (Guan et al., 2025). Long-term NPK fertilization further decreases rapeseed SMB diversity under stress conditions, as prolonged mineral inputs favor bacterial dominance while reducing overall microbial complexity and functional resilience (Dai et al., 2018; Li et al., 2021). In contrast, the application of manure combined with Bacillus species markedly increases microbial biomass (often enhancing activity and diversity through improved nutrient cycling), thereby bolstering drought tolerance via enhanced carbon and nitrogen transformations in the soil (Liang et al., 2025; Ng et al., 2025). Organic amendments thus preserve SMB functionality, nutrient turnover, enzyme activity, and stress-responsive guilds, directly augmenting oilseed resilience to escalating abiotic pressures (Kurzemann et al., 2020).

Organic fertilizers, by contrast, exert predominantly positive, sustainable effects on soil microbiomes. Application enhances crop yields, soil quality, and microbial diversity in temperate systems (Wang et al., 2025a). These amendments supply complex carbon substrates that stimulate microbial growth and foster guilds involved in nutrient cycling, decomposition, and aggregation. In soybean fields, organic fertilizers significantly increase bacterial abundance and reshape community composition, underscoring their role in microbial stimulation (Pan et al., 2025). Global meta-analyses confirm that organic systems deliver consistent environmental benefits, particularly for soil biological properties (Bocean, 2025). Although yield variability may rise, elevated microbial biomass and activity improve soil structure, water retention, and nutrient buffering (Holík et al., 2019). Long-term organic inputs boost enzyme activity and promote heterogeneous microbial distribution, enhancing ecosystem resilience under stress (Wang et al., 2024c). Organic amendments also improve physical properties indirectly supporting microbial function, yielding higher biomass and enzyme activity across depths, increased porosity, and better water retention during drought (Holík et al., 2019; Mo et al., 2024). Accumulated organic matter fosters diverse, functionally redundant communities.

Integrated strategies combining mineral and organic fertilizers balance immediate nutrient delivery with long-term soil health. Mineral inputs often produce variable, transient microbial effects, while organic components drive sustained diversity and activity gains. Soils receiving manure plus NPK exhibit elevated bacterial, actinobacterial, and gram-negative PLFA levels compared to mineral-only treatments, indicating greater biomass and community complexity (Mo et al., 2024). Partial replacement of chemical fertilizers with manure enhances diversity and restructures communities in vegetable systems, mitigating mineral fertilizer drawbacks via organic carbon supply and habitat improvement (Luan et al., 2020). Combined approaches elevate enzyme activity and bacterial diversity over mineral alone.

Integrated fertilization augments microbial functional potential, increasing enzymes for carbon, nitrogen, and phosphorus cycling processes vital for nutrient availability under stress, including water limitation or temperature extremes. Complementary mineral-organic roles thus sustain microbial networks resilient to abiotic perturbations (Liu et al., 2021b). Biofertilizers and microbial inoculants further enhance sustainable strategies by restructuring rhizosphere microbiomes and promoting disease suppression (Tsolakidou et al., 2019; Todorović et al., 2023). Plant growth-promoting microorganisms (PGPM) mitigate abiotic stress via improved nutrient uptake, phytohormone production, and physiological adaptation. For example, *Azospirillum* species enhance root development and uptake, supporting drought and nutrient deficiency tolerance (Naamala and Smith, 2020). Organic and integrated regimes create favorable conditions for PGPM proliferation. Humic substances from organic fertilizers also modulate microbial activity and plant-microbe interactions.

Fertilizer practices intersect with contamination and stressors. Heavy metal accumulation from intensive systems disrupts communities (Das et al., 2025). In *B. napus* soils, Cd/Ni contamination reduces enzymatic activity by 30–45%, favoring resistant *Bacillus* while depleting *Pseudomonas*. Organic amendments mitigate toxicity through immobilization and support of resistant populations (Boros-Lajszner et al., 2021).

Among strategies, organic amendments most effectively mitigate abiotic stress in oilseeds, increasing microbial biomass diversity by 40% and enzyme activity, yielding 18% higher rapeseed yields under drought and 32% improved sunflower salinity tolerance. Integrated NPK+organic ranks second, enhancing functional guilds and reducing metal bioavailability by 20% for multi-stress yield stability. Mineral NPK alone decreases diversity by 28% and phosphatase activity by 40%, compromising microbiome composition and imposing yield penalties under drought and salinity (Boros-Lajszner et al., 2021).

Organic fertilization thus emerges as the priority approach for enhancing abiotic stress resilience in oilseed crops, maximizing microbial functional redundancy and stress-responsive taxa essential for long-term sustainability amid climate change.

## 8 Conclusion and future directions

The complex bidirectional interactions between oilseed crops and their rhizosphere microbiome constitute a key area in the development of climate-resilient agricultural systems capable of withstanding intensifying abiotic stresses, including drought, salinity, heavy metal contamination, nutrient deficiencies, and extreme pH fluctuations. This review synthesizes compelling evidence that the rhizosphere microbiome acts as a dynamic buffer and modulator of plant stress responses, functioning as an adaptive interface that translates environmental cues into physiological and biochemical advantages for the host.

Across multiple studies, it has been consistently shown that abiotic stresses profoundly reshape soil physicochemical properties and microbial community assembly, yet stress-adapted microbial consortia maintain critical ecosystem functions—such as nutrient mineralization, phytohormone production, antioxidant enzyme induction, and improved water-use efficiency—that collectively enhance tolerance and yield stability in oilseed crops. Particular attention should be given to the Brassicaceae family (e.g., Brassica napus, B. juncea), where stress-induced activation of glucosinolate pathways, lipid-derived signals, and specific root exudation patterns selectively recruit beneficial taxa (e.g., Sphingomonadaceae, Bacillus, Pseudomonas, AMF) that confer cross-stress protection and support high seed oil content under adverse conditions.

Multi-omics integration (metagenomics, metatranscriptomics, metabolomics, and proteo-genomics) has significantly advanced mechanistic understanding by uncovering new unculturable stress-responsive guilds, functional redundancy networks, and genotype-specific microbiome signatures that underpin resilience under combined stresses (Yao et al., 2024; Yang et al., 2025b; Xing et al., 2021). Concurrently, agronomic management emerges as one of the most powerful levers: organic amendments, biofertilizers, reduced/no-tillage, and diversified rotations consistently promote microbial biomass, diversity, and functional potential, whereas prolonged reliance on intensive mineral fertilization and monoculture frequently leads to biodiversity loss, simplified interaction networks, and heightened vulnerability to abiotic perturbations.

Despite substantial progress in controlled environments, significant knowledge gaps persist at the field scale. Long-term, multi-site trials that simultaneously capture genotype × microbiome × management × climate interactions are still scarce, particularly in continental, arid, and semi-arid agroecosystems where oilseed crops are economically vital yet most vulnerable (Xing et al., 2021; Wassermann et al., 2022).

Future research directions should therefore include: (i) Large-scale, multi-year field experiments integrating high-resolution microbiome profiling with agronomic, physiological, and yield data across diverse edaphic and climatic gradients; (ii) Development and validation of synthetic or enriched microbial consortia (tailored SynComs) engineered for specific oilseed genotypes, soil types, and target stress combinations, with emphasis on long-term persistence, ecological stability, and non-target safety; (iii) Standardization of protocols for assessing inoculant establishment, functional expression *in situ*, and legacy effects on native microbiome communities; (iv) Exploration of genotype-microbiome co-selection breeding strategies, including identification of plant loci that control beneficial microbiome recruitment under stress; (v) Incorporation of predictive modeling (machine learning, network analysis) to forecast microbiome responses to management practices and climate scenarios, facilitating precision agronomy.From an economic perspective, microbiome-based strategies offer the potential to reduce reliance on chemical fertilizers and pesticides, lower input costs, and improve resource-use efficiency in oilseed production systems. Enhanced nutrient acquisition and stress resilience may contribute to more stable yields under variable climatic conditions, which is particularly important for risk-prone arid and semi-arid regions. However, the economic feasibility of these approaches depends on several factors, including the cost of inoculant production, formulation stability, field-level performance consistency, and ease of integration into existing farming practices. Large-scale validation and cost–benefit analyses are therefore essential to support farmer adoption and ensure the practical viability of microbiome-driven agricultural technologies.

Ultimately, harnessing the full potential of the plant genotype–rhizosphere microbiome–environment triad through integrated, sustainable agronomic frameworks will be crucial for building resource-efficient, high-yielding oilseed production systems resilient to future climate variability and supporting global food and bioenergy security in continental agroecosystems.

## Glossary

PGPR: Plant Growth Promoting Rhizobacteria
SynComs: Synthetic Microbial Communities
IAA: Indole-3-acetic acid
GA: Gibberellic acid
ACC-desaminases: 1-aminocyclopropane-1-carboxylic acid deaminase
ROS: Reactive Oxygen Species
AMF: Arbuscular Mycorrhizal Fungi
CAT: Catalase
SOD: Superoxide Dismutase
POD: Peroxidase
GR: Glutathione Reductase
GPX: Glutathione Peroxidase
ABA: Abscisic Acid
HSPs: Heat Shock Proteins
NGS: Next-Generation Sequencing
PLFA: Phospholipid Fatty Acids
EPS: Exopolysaccharides
